# Temporomandibular disorders patients with migraine symptoms have increased disease burden due to psychological conditions

**DOI:** 10.22514/jofph.2025.006

**Published:** 2025-03-12

**Authors:** Soo Haeng Lee, Jung Hwan Jo, Ji Woon Park

**Affiliations:** ^1^Department of Oral Medicine, Seoul National University Dental Hospital, 03080 Seoul, Republic of Korea; ^2^Department of Oral Medicine and Oral Diagnosis, Seoul National University School of Dentistry, 03080 Seoul, Republic of Korea; ^3^Dental Research Institute, Seoul National University, 03080 Seoul, Republic of Korea

**Keywords:** Temporomandibular disorders, Migraine, Sumatriptan, Psychological problem, Disease burden

## Abstract

**Background**: Various studies have demonstrated a close link between headaches and temporomandibular disorders (TMD). However, the results are often limited to certain clinical aspects and are based on a cross-sectional study design. This study aimed to examine the clinical characteristics of patients with both TMD and migraine symptoms and to assess the long-term treatment outcomes compared to TMD patients without migraine. **Methods**: Sixty-four TMD patients were evaluated using the Diagnostic Criteria for TMD protocol and validated questionnaires, including Generalized Anxiety Disorder-7, Patient Health Questionnaire (PHQ)-9, PHQ-15, the Graded Chronic Pain Scale, and the Symptom Checklist-90-Revision (SCL-90-R). Patients were divided into two groups based on the presence of migraine symptoms requiring medication. The study compared psychological and clinical profiles, as well as long-term treatment outcomes. **Results**: The migraine group exhibited greater psychological distress, as indicated by higher scores in the SCL-90-R subscales for somatization (*p* = 0.035), obsessive-compulsive behavior (*p* = 0.015), interpersonal sensitivity (*p* = 0.002), depression (*p* = 0.035), anxiety (*p* = 0.042), hostility (*p* = 0.004), paranoid ideation (*p* = 0.016), and psychoticism (*p* = 0.044). Additionally, they scored higher on the PHQ-9 (*p* = 0.023) and PHQ-15 (*p* = 0.016). Pain levels were higher in the migraine group at 3 months post-treatment (*p* = 0.023) but the difference with the non-migraine group disappeared 6 months post-treatment. Younger age (odds ratio (OR) = 0.844, *p* = 0.001), female (OR = 0.001, *p* = 0.011), and more positive sites on masticatory muscle palpation (OR = 2.580, *p* = 0.011) were associated with a higher likelihood of experiencing migraine. Mental illness history (β = −0.465, *p* = 0.002), tongue ridging (β = −0.683, *p* < 0.001), and Oral Behavior Checklist scores (β = 0.483, *p* = 0.002) were associated with TMD pain intensity in the migraine group. **Conclusions**: TMD patients using sumatriptan for migraine symptoms had higher levels of disability and psychological distress, leading to an increased disease burden. Although the migraine group had worse short-term TMD treatment outcomes, these differences resolved after six months of treatment.

## 1. Introduction

Temporomandibular disorders (TMD) is a term used to refer to musculoskeletal and neuromuscular disorders involving the temporomandibular joint (TMJ), muscles of mastication, and related structures [1]. TMD could be divided into three pain related groups which are muscle disorders such as myofascial pain, arthralgia, and headache attributed to TMD. Also, intra-articular disorders such as disc displacement with or without reduction and mouth opening limitation exist along with degenerative joint disorder and subluxation [2]. The prevalence of TMD is 5–12% of the adult population making it the most common cause of non-odontogenic pain in the orofacial area [3]. At the same time, TMD is the second most common musculoskeletal disorder that causes considerable pain and disability [4, 5]. Symptoms include reduced range of jaw motion, pain in the masticatory muscles and joint, joint noise, and deviation in mouth opening. Additionally, patients may experience symptoms such as ear pain, tinnitus, vertigo, and headaches [6]. While symptoms are not life-threatening, it may run a chronic course impairing quality of life significantly [7].

Migraine is known as the most common headache in TMD patients [8]. The prevalence of any migraine in TMD patients increased from 16.1% to 28% compared to the 8.4% to 12% in TMD free controls after 3 years. Furthermore, for definite migraine, this number increased approximately 10 times from 1.2% to 9.7% in TMD patients [9]. Patients with migraine had a significantly higher prevalence of muscular TMD compared to patients without migraine while this was not true for those with joint related TMD. On the other hand, another investigation showed that patients with muscular TMD had a significantly higher prevalence of migraine compared to patients without muscle related TMD [10]. Limited lateral jaw movement, joint sounds, and neck muscle tenderness were also observed more frequently in migraine patients [11]. As a subtype of migraine, facial migraine is defined as episodic or chronic pain localized to the orofacial region without head pain however, sharing the clinical characteristics of migraine [12]. Among migraine patients approximately 9% reported symptoms that extended to the lower face. Reports on isolated facial migraine are rare and often subject to selection bias due to ambiguity regarding pain localization [13]. A previous study showed that among 409 facial pain patients, 51 were diagnosed with migraine and 24 of these cases had isolated pain of the second division of the trigeminal nerve [14]. In a study with a larger sample size of 1176 patients with migraine, isolated facial pain was seen in 4.9% [15]. In another study of 1935 migraine patients 2.3% experienced facial pain while 40.9% of these patients reported pain which was concentrated in the face area [16]. Triptan is known to effectively control moderate to severe migraine as a first-line treatment [17, 18]. It is a 5-hydroxytryptamine receptor agonist and the most commonly used migraine-specific medication, with minimal major side effects when taken at appropriate doses [18].

Although substantial evidence highlights the intimate and complex relationship between TMD and migraine, studies are often limited to analyzing symptom characteristics, overlooking the psychological status and interference level experienced by patients suffering from both conditions simultaneously.

Therefore, the main objective of this study was to analyze clinical characteristics, specifically psychological factors and resulting disability of TMD patients prescribed migraine medication (sumatriptan) due to migraine symptoms in a cross-sectional manner. Additionally, the study aimed to compare long-term treatment outcomes between the two groups with particular attention to the increased disease burden posed by the presence of migraine in a well-defined TMD population.

## 2. Materials and methods

### 2.1 Subjects

This retrospective study involved those who visited the Department of Oral Medicine of Seoul National University Dental Hospital with the chief complaint of pain and dysfunction in the TMJ and surrounding area from September 2019 to December 2023. Exclusion criteria were patients who had not received conservative treatment for TMD or those with missing clinical information among the observational items. For the control group, 32 consecutive patients who were diagnosed as TMD but did not report migraine symptoms during the same period were randomly selected from the patient visit record. All methods were performed in accordance with the Declaration of Helsinki and relevant guidelines. This work was approved by the Institutional Review Board of Seoul National University Dental Hospital (ERI24003). All patients provided informed written consent to usage of their clinical data for academic purposes on their first visit to the hospital. Waiver of additional informed consent was granted based on the retrospective nature of the study.

### 2.2 Assessment of temporomandibular disorders and related comorbidities

TMD was diagnosed following the diagnostic criteria for TMD (DC/TMD) [2]. All physical examinations were done by a single experienced orofacial pain specialist. Clinical parameters included comfortable (CMO) and maximum mouth opening (MMO) range. Pain on palpation of the masticatory and cervical muscles was assessed with positive sites counted out of 4 sites respectively, as well as the 2 TMJ capsule areas from both sides. Additionally, pain on mouth opening and eccentric mandibular movements (protrusion and laterotrusion) were examined. Subjective pain intensity was evaluated on a numeric rating scale (NRS) of 0 to 10. Other parameters such as tooth attrition, tongue ridging, and mucosal ridging were also recorded. Degenerative joint disease was diagnosed based on panoramic, transcranial, and TMJ panoramic radiographs by verifying the presence of erosion, osteophyte and/or subcortical cyst of the TMJ condylar head.

A comprehensive interview was conducted concerning demographic features, general medical condition, and comorbidities including sleep disturbance, neck and shoulder pain, lower back pain, arm and leg pain, and gastrointestinal disorder. A headache questionnaire was used to assess the patient’s headache experience [19]. The presence of migraine was diagnosed by the same single orofacial pain specialist with more than 15 years of clinical experience based on patient reports of migraine symptoms including pulsating nature of face and/or head area pain, concomitant nausea and/or vomiting. When the patient reported headache symptoms that did not match the diagnostic criteria for migraine, sumatriptan was not prescribed.

Psychological status and disability levels were evaluated in all patients with DC/TMD axis II questionnaires including GAD-7, PHQ-9, PHQ-15 and the Graded Chronic Pain Scale [2] and Symptom Checklist-90-Revision (SCL-90-R) test [20].

### 2.3 Assessment of long-term treatment response

Conservative treatment included cognitive and behavioral therapy such as control of contributing factors and self-exercise, occlusal stabilization splint, physical therapy including moist hot pack, ultrasound, electrical stimulation and low-level laser, and non-steroidal anti-inflammatory drugs.

Patients were re-evaluated for CMO, MMO, pain on palpation of masticatory muscles and TMJ capsule, pain on mouth opening and pain intensity by the same clinician and data from 3 and 6 months from the first examination were gathered.

### 2.4 Statistical analysis

Normality of data was tested with Kolmogorov-Smirnov test and analysis methods were selected accordingly. Differences between migraine medication and non-migraine medication groups were analyzed with student’s *t*-test or Mann-Whitney U test and chi-square test, Fisher’s exact test or Linear by linear association. Changes in clinical signs at 3 and 6 months were analyzed with generalized estimating equations. In the generalized estimating equations, the chi-square data of continuous and categorical variables were adjusted for compound level clustering by Poisson log-linear and logistic regression analysis, respectively. Clinical variables associated with migraine in TMD patients were analyzed with logistic regression. Clinical variables associated with TMD pain intensity were analyzed with multiple regression analysis. All statistical analyses were performed using SPSS 26.0 software (IBM, Chicago, IL, USA). Level of statistical significance was set at *p* < 0.05.

## 3. Results

### 3.1 Clinical characteristics according to concomitant migraine

The study involved 64 consecutive participants (mean age 41.4 ± 2.1 years, 57 women and 7 men).

During the study period, 14,741 patients visited with TMD symptoms as their chief complaint and were subsequently diagnosed as TMD. Among these patients, 40 were prescribed sumatriptan due to their migraine symptoms. After excluding those with insufficient data at 6 months follow-up, 32 patients were included in the final analysis.

As presented in Table 1, the mean age was significantly lower in the migraine group compared to the non-migraine group (*p* = 0.001). The duration of TMD pain was shorter in the migraine group compared to the non-migraine group (*p* = 0.056). Closed locking of the jaw was significantly more frequent in the migraine group than in the non-migraine group (*p* = 0.017). Mucosal ridging was significantly more prevalent in the non-migraine group compared to the migraine group (*p* = 0.032). The high score group based on the Oral Behavior Checklist, indicative of parafunctional behavior, was significantly more prevalent in the migraine group (*p* = 0.044).

**Table 1.** Clinical characteristics according to concomitant migraine.

Variable		Migraine (n = 32)	Non-migraine (n = 32)	*p*
Age (yr)^a^		34.9 (2.47)	47.9 (2.91)	0.001*
Gender (M/F)^b^		2/30	5/27	0.426
Medical history^b^				
	Hypertension	0/32 (0.0%)	6/32 (18.8%)	0.012*
	Hyperlipidemia	3/32 (9.4%)	10/32 (31.3%)	0.030*
	Mental illness	5/32 (15.6%)	2/32 (6.3%)	0.213
TMD pain duration (months)^c^		30.0 (5.3, 84.0)	96.0 (10.5, 246.8)	0.056
Jaw joint noises^b^		24/27 (88.9%)	24/30 (80.0%)	0.476
Closed locking of the jaw^d^		17/27 (63.0%)	9/30 (30.0%)	0.017*
Open locking of the jaw^b^		5/25 (20.0%)	5/29 (17.2%)	1.000
CMO (mm)^c^		35.50 (30.75, 49.50)	40.00 (34.25, 48.00)	0.493
MMO (mm)^c^		44.50 (40.00, 50.00)	45.00 (41.25, 50.00)	0.618
Masticatory muscle palpation (positive sites out of 4)^c^		2.00 (1.00, 4.00)	1.50 (0.00, 4.00)	0.096
Cervical muscle palpation (positive sites out of 4)^c^		1.00 (0.00, 2.00)	0.00 (0.00, 1.00)	0.103
Capsule palpation (positive sites out of 2)^c^		1.00 (0.00, 1.00)	0.00 (0.00, 2.00)	0.714
Pain intensity (0–10 NRS)^c^		7.00 (5.13, 8.00)	5.00 (3.50, 8.00)	0.121
Tooth attrition^d^		6/31 (19.4%)	12/32 (37.5%)	0.164
Tongue ridge^d^		19/31 (61.3%)	27/32 (84.4%)	0.050
Mucosal ridge^d^		21/31 (67.7%)	29/32 (90.6%)	0.032*
Pain on mouth opening^d^		20/32 (62.5%)	13/32 (40.6%)	0.080
Pain on Eccentric movement^d^		11/30 (36.7%)	10/32 (31.3%)	0.789
DJD^b^		10/12 (83.3%)	14/14 (100.0%)	0.203
Jaw Function Limitation Scale-20 (0–10 NRS)				
	Mastication^a^	3.51 (0.43)	3.36 (0.46)	0.818
	Mobility^a^	3.34 (0.45)	2.65 (0.40)	0.259
	Verbal and non-verbal communication^c^	1.98 (1.16, 2.80)	1.36 (0.70, 2.03)	0.277
Oral Behavior Checklist^e^				
	None (score 0)	1/32 (3.1%)	5/32 (15.6%)	0.044*
	Low (score 1–24)	21/32 (65.6%)	22/32 (68.8%)	
	High (score 25–84)	10/32 (31.3%)	5/32 (15.6%)	

CMO: comfortable mouth opening; MMO: maximum mouth opening; NRS, numeric rating scale; DJD: degenerative joint disease of the temporomandibular joint; M/F: male/female; TMD: temporomandibular disorders. ^a^Student’s t-test: mean (standard deviation, SD). ^b^Fisher’s exact test. ^c^Mann-Whitney U test: Median (lower quartile, upper quartile). ^d^Chi-square test: number of positive subjects. ^e^Linear by linear association. *Significant difference, p < 0.05.

### 3.2 Headache characteristics according to concomitant migraine

As shown in Table 2, clenching, grinding, or chewing gum was reported significantly more often in those with migraine (*p* = 0.026). In the migraine group, throbbing sensation was significantly more frequent than in the non-migraine group (*p* = 0.021). The presence of nausea was significantly more prevalent in the migraine group than in the non-migraine group (*p* < 0.001). Unilaterality of headache was not prominent in the migraine group.

**Table 2.** Headache characteristics according to concomitant migraine.

Variable		Migraine (n = 32)	Non-migraine (n = 32)	*p*
Headache (yes/no)^a^		23/26 (88.5%)	29/30 (96.7%)	0.328
Headache duration (mon)^b^		12.0 (2.0, 84.0)	120.0 (16.5, 271.8)	0.127
Contributing factors of headache^c^				
	Chewing hard or tough food	13/22 (59.1%)	12/31 (38.7%)	0.143
	Jaw movement	12/22 (54.5%)	12/31 (38.7%)	0.254
	Clenching/grinding, chewing gum	16/22 (72.7%)	13/31 (41.9%)	0.026*
	Other jaw activities (talking, kissing, or yawning)	12/22 (54.5%)	9/31 (29.0%)	0.061
Characteristics^c^				
	Pressing/tightening	24/32 (75.0%)	19/32 (59.4%)	0.183
	Pulsating/throbbing	27/32 (84.4%)	18/31 (58.1%)	0.021*
Location^c^				
	Unilateral	13/30 (43.3%)	18/31 (58.1%)	0.310
	Bilateral	17/30 (56.7%)	13/31 (41.9%)	
Aggravated by routine activity^c^		7/32 (21.9%)	6/31 (19.4%)	0.805
Severity^d^				
	Mild (NRS ≤3)	4/32 (12.5%)	10/32 (31.3%)	0.090
	Moderate (NRS 4–6)	9/32 (28.1%)	8/32 (25.0%)	
	Severe (NRS ≥7)	19/32 (59.4%)	14/32 (43.8%)	
Nausea, vomiting, photophobia, phonophobia^c^		31/32 (96.9%)	15/32 (46.9%)	<0.001*
Previous medication^a^				
	Acetaminophen	8/14 (57.1%)	6/10 (60.0%)	1.000
	NSAIDs	12/15 (80.0%)	7/11 (63.6%)	0.407

NRS: numeric rating scale; NSAIDs: nonsteroidal anti-inflammatory drugs. ^a^Fisher’s exact test. ^b^Mann-Whitney U test: Median (lower quartile, upper quartile). ^c^Chi-square test: number of positive subjects. ^d^Linear by linear association. *Significant difference, p < 0.05.

### 3.3 Psychological characteristics and disability levels according to concomitant migraine 

As shown in Table 3, participants in the migraine group reported significantly higher scores in most subcategories and global scores of the SCL-90-R including somatization (*p* = 0.035), obsessive-compulsive (*p* = 0.015), interpersonal sensitivity (*p* = 0.002), depression (*p* = 0.035), anxiety (*p* = 0.042), hostility (*p* = 0.004), paranoid ideation (*p* = 0.016), psychoticism (*p* = 0.044), global severity index (*p* < 0.001), and positive symptom score (*p* = 0.032) compared to the non-migraine group. Additionally, PHQ-9 (*p* = 0.023) and PHQ-15 (*p* = 0.016) scores showed significant differences, indicating higher levels of depression and somatic symptoms in the migraine group.

**Table 3.** Psychological characteristics according to concomitant migraine.

Variable		Migraine (n = 32)	Non-migraine (n = 32)	*p*
SCL-90-R				
	Somatization^a^	52.5 (1.52)	48.4 (1.13)	0.035*
	Obsessive-compulsive^a^	46.7 (1.87)	41.1 (1.19)	0.015*
	Interpersonal sensitivity^b^	46.8 (43.9, 49.6)	41.1 (38.8, 43.3)	0.002*
	Depression^b^	47.8 (44.6, 50.9)	43.4 (40.8, 46.0)	0.035*
	Anxiety^b^	47.9 (44.3, 51.5)	43.3 (41.2, 45.5)	0.042*
	Hostility^b^	46.6 (44.2, 48.9)	42.2 (40.9, 43.5)	0.004*
	Phobic anxiety^b^	47.4 (43.9, 51.0)	44.8 (42.7, 46.8)	0.244
	Paranoid ideation^b^	44.6 (42.5, 46.8)	42.3 (39.1, 45.4)	0.016*
	Psychoticism^b^	45.5 (43.2, 47.8)	43.0 (40.7, 45.3)	0.044*
	Global severity index^a^	47.5 (1.48)	41.4 (0.92)	<0.001*
	Positive symptom distress index^b^	46.5 (41.0, 51.5)	43.0 (39.0, 50.0)	0.152
	Positive symptom total^a^	47.7 (1.65)	42.5 (1.74)	0.032*
GAD-7^c^				
	None (sum 0–4)	14/32 (43.8%)	21/32 (65.6%)	0.068
	Mild anxiety (sum 5–9)	11/32 (34.4%)	8/32 (25.0%)	
	Moderate anxiety (sum 10–14)	4/32 (12.5%)	2/32 (6.3%)	
	Severe anxiety (sum 15–21)	3/32 (9.4%)	1/32 (3.1%)	
PHQ-9^c^				
	None (sum 0–4)	10/32 (31.3%)	19/32 (59.4%)	0.023*
	Mild depression (sum 5–9)	14/32 (43.8%)	11/32 (34.4%)	
	Moderate depression (sum 10–14)	4/32 (12.5%)	1/32 (3.1%)	
	Moderate-to-severe depression (sum 15–19)	3/32 (9.4%)	0/32 (0.0%)	
	Severe depression (sum 20–27)	1/32 (3.1%)	1/32 (3.1%)	
PHQ-15^c^				
	None (Sum 0–4)	7/32 (21.9%)	12/32 (37.5%)	0.016*
	Low symptom severity (sum 5–9)	10/32 (31.3%)	15/32 (46.9%)	
	Medium symptom severity (sum 10–14)	9/32 (28.1%)	3/32 (9.4%)	
	High symptom severity (sum 15–30)	6/32 (18.8%)	2/32 (6.3%)	
Graded Chronic Pain Scale^c^				
	Grade 0	0/30 (0.0%)	1/28 (3.6%)	0.092
	Grade I	7/30 (23.3%)	10/28 (35.7%)	
	Grade II	4/30 (13.3%)	5/28 (17.9%)	
	Grade III	8/30 (26.7%)	6/28 (21.4%)	
	Grade IV	11/30 (36.7%)	6/28 (21.4%)	

GAD-7: Generalized Anxiety Disorder; PHQ: Patient Health Questionnaire; SCL-90-R: Symptom Checklist-90-Revision. ^a^Student’s t-test: mean (SD). ^b^Mann-Whitney U test: Median (lower quartile, upper quartile). ^c^Linear by linear association. *Significant difference, p < 0.05.

### 3.4 Long-term temporomandibular disorders symptom change according to concomitant migraine

As shown in Table 4, the difference in pain intensity before TMD treatment was not statistically significant between the 2 groups. At 3 months post-treatment, both groups showed a significant decrease in pain intensity, however pain levels were significantly higher in the migraine group (*p* = 0.023). By six months post-treatment, pain intensity had decreased further in both groups however, while the pain level remained higher in the migraine group, the difference was no longer statistically significant. Significant changes over time and between groups were observed with significant interaction between visit and group.

**Table 4.** Long-term change in TMD symptoms according to concomitant migraine.

Group		Initial visit	3-month	6-month	χ^2^		
					Visit	Group	Visit × Group
Pain intensity (NRS)^a^							
	Migraine	7.00 (5.13, 8.00)	6.75 (3.63, 7.75)	4.00 (0.00, 6.25)	33.041**	11.041**	6.808*
	Non-migraine	5.00 (3.50, 8.00)	3.00 (0.00, 6.63)	3.50 (0.00, 7.00)			
	*p*	0.121	0.023*	0.649			
CMO (mm)^a^							
	Migraine	35.50 (30.75, 49.50)	40.00 (33.75, 50.25)	40.00 (38.00, 52.25)	16.633**	1.521	0.184
	Non-migraine	40.00 (34.25, 48.00)	43.50 (37.25, 49.75)	46.00 (42.50, 50.00)			
	*p*	0.493	0.537	0.860			
MMO (mm)^a^							
	Migraine	44.50 (40.00, 50.00)	44.00 (39.50, 50.25)	46.00 (41.00, 52.50)	1.825	0.192	0.168
	Non-migraine	45.00 (41.25, 50.00)	45.00 (40.50, 51.75)	46.00 (43.00, 50.00)			
	*p*	0.618	0.614	0.930			
Pain on masticatory muscle palpation^b^							
	Migraine	26/32 (81.3%)	16/22 (72.7%)	13/21 (61.9%)	3.751	1.079	1.882
	Non-migraine	19/32 (59.4%)	15/20 (75.0%)	11/17 (64.7%)			
	*p*	0.099	1.000	1.000			
Pain on capsule palpation^b^							
	Migraine	17/32 (53.1%)	9/22 (40.9%)	7/21 (33.3%)	6.018*	1.030	0.082
	Non-migraine	14/32 (43.8%)	7/20 (35.0%)	5/17 (29.4%)			
	*p*	0.617	0.758	1.000			
Pain on mouth opening^b^							
	Migraine	20/32 (62.5%)	9/22 (40.9%)	7/21 (33.3%)	12.091*	1.669	0.676
	Non-migraine	13/32 (40.6%)	5/20 (25.0%)	5/17 (29.4%)			
	*p*	0.080	0.338	1.000			

NRS: numeric rating scale; CMO: comfortable mouth opening; MMO: maximum mouth opening. ^a^Differences between groups were tested with Mann-Whitney U test: median (25%, 75%); chi-square data were adjusted for compound level clustering by generalized estimating equations (GEE) Poisson log-linear analysis. ^b^Differences between groups were tested with chi-square test: number of subjects (%); chi-square data were adjusted for compound level clustering by GEE logistic regression analysis. *Significant difference, p < 0.05, **Significant difference, p < 0.001.

CMO and MMO increased over time in both groups. Significant changes over time were observed for CMO but not for MMO.

The percentage of patients reporting pain on masticatory muscle and TMJ capsule palpation decreased in both groups. Significant changes over time were observed for capsule palpation (*p* = 0.049) but not for masticatory muscle palpation. The percentage of patients reporting pain on mouth opening was higher in the migraine group and decreased in both groups with treatment. The change was significant over time (*p* = 0.002).

### 3.5 Clinical characteristics associated with migraine and TMD pain intensity

As shown in Table 5, age was significantly associated with migraine with younger patients more likely to report migraine symptoms (odds ratio (OR) = 0.844, *p* = 0.001). Female gender also showed a significant association with migraine (OR = 0.001, *p* = 0.011). TMD patients with more positive sites on masticatory muscle palpation were associated with migraine (OR = 2.580, *p* = 0.011).

**Table 5.** Clinical variables associated with concomitant migraine in temporomandibular disorders.

Variable	β	Standard error	OR	95% CI (lower–upper)	*p*
Age (yr)	−0.170	0.053	0.844	0.761–0.935	0.001*
Gender	−7.046	2.786	0.001	0.000–0.205	0.011*
Mental illness	0.586	1.417	1.796	0.112–28.899	0.679
CMO	0.025	0.077	1.025	0.881–1.193	0.746
MMO	−0.087	0.107	0.917	0.743–1.132	0.419
Masticatory muscle palpation (positive sites out of 4)	0.948	0.372	2.580	1.245–5.347	0.011*
Cervical muscle palpation (positive sites out of 4)	−0.307	0.455	0.736	0.302–1.794	0.500
Capsule palpation (positive sites out of 2)	−0.824	0.780	0.439	0.095–2.025	0.291
NRS	−0.093	0.208	0.911	0.605–1.370	0.654
Attrition	−0.481	1.176	0.618	0.062–6.192	0.682
Tongue ridge	−1.173	1.659	0.309	0.012–8.000	0.480
Mucosal ridge	−4.333	2.223	0.013	0.000–1.025	0.051

The results were obtained from logistic regression analysis. CMO: comfortable mouth opening; MMO: maximum mouth opening; NRS: numeric rating scale; OR: odds ratio; CI: confidence interval. Reference group for statistical comparisons: Sex variable is based on female and the others are based on negative response. *Significant difference, p < 0.05.

As shown in Table 6, mental illness history (β = −0.465, *p* = 0.002) and tongue ridging (β = −0.683, *p* < 0.001) showed a negative association while high score on the Oral Behavior Checklist (β = 0.483, *p* = 0.002) showed positive association with TMD pain intensity in the migraine group. In the non-migraine group, tooth attrition (β = −0.364, *p* = 0.029) showed a negative association, whereas the number of positive sites on masticatory muscle palpation (β = 0.398, *p* = 0.019) was positively associated with TMD pain intensity.

**Table 6.** Clinical variables associated with TMD pain intensity according to concomitant migraine.

Variable	Migraine				Non−migraine			
	Coefficient	β	95% CI	*p*	Coefficient	β	95% CI	*p*
Age	−0.001	−0.009	−0.051–0.048	0.960	0.040	0.226	−0.027–0.108	0.232
Gender	−0.430	−0.054	−2.732–1.872	0.702	−0.596	−0.074	−3.330–2.138	0.657
Mental illness	−2.715	−0.465	−(4.310)–(–1.120)	0.002*	0.967	0.081	−3.331–5.266	0.647
Hyperlipidemia	1.418	0.214	−1.000–3.835	0.236				
Capsule palpation	−0.203	−0.099	−0.946–0.539	0.575				
Masticatory muscle palpation	−0.069	−0.095	−0.428–0.291	0.695	0.466	0.398	0.085–0.847	0.019*
Cervical muscle palpation	0.102	0.079	−0.339–0.544	0.635				
Tongue ridge	−2.746	−0.683	−(3.954)–(–1.537)	<0.001*				
Tooth attrition					−2.185	−0.364	−(4.130)–(–0.240)	0.029*
Hypertension					−0.254	−0.034	−3.102–2.595	0.856
Mucosal ridge					−2.286	−0.229	−5.813–1.241	0.194
OBC_high	2.023	0.483	0.860–3.186	0.002*				

The results were obtained from multiple regression analysis. OBC: Oral Behavior Checklist; CI: confidence interval. Reference group for statistical comparisons: Sex variable is based on female, OBC is based on none and the others are based on negative response. *Significant difference, p < 0.05.

## 4. Discussion

The results of this study revealed that TMD patients with accompanying migraine symptoms experienced higher levels of disability and psychological issues, leading to an increased overall disease burden. Furthermore, these patients exhibited worse short-term outcomes after conventional TMD treatment compared to those without migraine however, this difference in TMD symptoms was overcome by long-term conventional treatment. These findings suggest that the presence of migraine symptoms requiring sumatriptan may influence the prognosis of TMD and introduce additional challenges for this patient group, with effects that vary depending on the duration of treatment.

A previous study showed that migraine prevalence was higher in younger age groups, specifically in those in their 30 s, with a prevalence of 20.13% [21]. This is consistent with our study which shows a similar prevalence of 21.88% for the same age group indicating the need to probe for headache related symptoms in this specific age group of TMD patients.

The prevalence of hypertension and hyperlipidemia showed a significant difference according to migraine group. According to previous studies, there was no significant correlation between the presence of metabolic syndrome and migraine, presenting conflicting results [22]. Such results should be interpreted while considering the significant age difference between the two groups and the positive relationship between age and hypertension, as well as hyperlipidemia [23].

In this study, the duration of TMD was shorter in the migraine group compared to the non-migraine group. Patients with headaches may recognize TMD pain at an earlier stage due to increased awareness of the orofacial region and take appropriate measures reducing the duration of pain. Although TMD is known to be more prevalent in the 20–40 s age group, due to the older average age of the non-migraine group the patients of this group may recall the duration of their symptoms as being longer due to the typical wax and wane pattern of chronic disorders. Another study found that individuals with migraines experienced a longer average duration of TMD (89.3 months) compared to those with tension-type headaches (78.8 months) or no headaches (72.8 months), though the difference was not statistically significant [24].

Interestingly, mucosal ridging was more prevalent in the non-migraine group. Ridging of the buccal mucosa has been repeatedly noted in those with clenching as a reliable diagnostic sign. However, since swallowing occurs throughout the day, the repeated pressure exerted by the buccal surface of teeth during swallowing might contribute to the formation of buccal mucosa ridging irrelevant of clenching. Also, it has been suggested that the pressure applied on the buccal mucosa by the tooth surface is not significantly associated with the electromyographic activity level of the masseter and buccinator muscles [25]. On the other hand, clenching and grinding as parafunctional habits were more prevalent in the migraine group. Also, according to multiple regression analyses, an increase in parafunctional behaviors was positively associated with TMD pain. This aligns with previous studies indicating that oral parafunctional habits such as bruxism, jaw thrusting, chin cupping, and resting the hand on the side of the face are more common in those with migraine [26]. A recent preclinical investigation showed that mimicking TMD pain by injecting an algesic/inflammatory mediator into the masseter muscle effectively activated and sensitized trigeminal neurons. These neurons receive nociceptive inputs from both dural-intracranial and cutaneous-extracranial sources. The activation of these neurons strongly resembles headache pain, suggesting the convergence of trigeminal and intracranial neurons related to headache and mediation of headache-like responses directly [8]. Also, this relationship is bidirectional, with worsening TMD symptoms leading to more frequent and severe headaches, and increasing headache severity intensifying TMD symptoms [8, 27, 28, 29]. This interaction implies a reduced threshold for triggering headache-like symptoms in the intracranial area [8]. Patients with TMD are often known to exhibit symptoms that overlap with other chronic pain conditions, such as fibromyalgia and certain neurological disorders. This may be due to central sensitization, a phenomenon characterized primarily by clinical features like allodynia and hyperalgesia [28].

One study reported results based on SCL-90-R in migraine patients indicating higher scores in somatization, anxiety, depression, anger, interpersonal sensitivity, phobia, paranoia, psychotic symptoms, and anger subscales compared to control groups [30]. Another study found that depression and anxiety were the most prevalent comorbid psychological disorders [31]. The elevated psychological conditions found in the migraine group in this study are consistent with these results. The results showed that depression levels based on PHQ-9 scores were higher in the migraine group as in other studies [32, 33].

Additionally, the higher frequency of somatic symptoms in the migraine group supports previous research showing that the severity of somatic symptoms is associated with headache frequency [34]. The following evidence may explain the mechanism linking migraine and mental instability. First, the serotonin (5-HT) system is essential in the relationship between migraine and depression [35, 36, 37]. Typically, migraine patients experience elevated 5-HT levels during attacks. Over time, the availability of 5-HT between attacks tends to diminish, which is believed to be linked to worsening depression and heightened sensitivity of the trigemino-vascular pathway [38]. Additionally, 5-HT receptor gene polymorphism can affect not only migraine but also depression [35, 39]. The second most important role is played by the dopamine system. Dopamine signaling is influenced by the dopamine receptor D2 (DRD2) NcoI C/C genotype [35, 37, 40] which shows a significant association with migraine, depression, and anxiety [41]. Another possibility is that significantly lower gamma-aminobutyric acid (GABA) cerebrospinal fluid levels may be significantly associated with both depression and migraine. The other possibility is common involvement of the hypothalamic-pituitary-adrenal (HPA) axis. It is hypothesized that an imbalance between pro-inflammatory cytokines and anti-inflammatory cytokines may result in dysfunction of tryptophan metabolism and serotonin activation in the HPA axis [35, 36, 37].

Migraine is often accompanied by various mental disorders, but such disorders are frequently undiagnosed and untreated [42]. One of the most important factors contributing to this is the fear of stigmatization [43]. Additionally, the migraine group showed a tendency for reporting higher levels of limitation to daily activities reflecting the overall increase in disease burden with concomitant migraine headache. Psychological treatments, including cognitive behavioral therapy, behavior modification therapy, mindfulness and when necessary, interventions from specialists are strongly recommended for chronic TMD patients [44]. Clinicians may need to consider more proactive psychological approaches when treating TMD patients who exhibit migraine symptoms. This approach can assist clinicians in providing better support to patients who may feel discouraged by the absence of immediate improvement.

At 3-month post-treatment, both groups experienced a significant reduction in pain intensity, demonstrating the effectiveness of conventional TMD treatment. However, the group with migraine reported significantly higher pain levels compared to the non-migraine group. This suggests that while TMD treatment is beneficial for both groups, patients with migraine may experience less effective pain relief with TMD treatment. TMD and migraine share trigeminal nerve activation [8] and may exacerbate each other bidirectionally [8, 27, 28, 29]. Additionally, heightened levels of psychological conditions are observed in the migraine group and the insufficient improvement of such problems may worsen both conditions. At 6-month post-treatment, both groups showed further reduction in pain intensity, indicating the long-term benefits of TMD treatment regardless of the presence of headache. Notably, although pain levels remained higher in the migraine group the statistical significance of this difference disappeared. This suggests that while the initial response to TMD treatment may be influenced by the presence of migraine, the long-term benefits tend to converge between the two groups.

Masticatory muscle palpation showed a significant positive association with migraine, which is consistent with previous studies indicating that patients with migraine have a significantly higher prevalence of muscular TMD compared to those without migraine [10]. Studies highlight that pre-existing myogenic TMD exacerbates nitroglycerin-induced hypersensitivity as in migraine through upregulation of calcitonin gene related peptide (CGRP) in the spinal trigeminal nucleus caudalis, suggesting the need for integrated treatment approaches for TMD and migraine due to their shared pathophysiological pathways [45].

Several limitations should be considered when interpreting the results of this study. First, the relatively small sample size limits the ability to draw strong and broadly applicable conclusions. Future studies with larger cohorts will be needed to address this issue. There are inherent issues due to the retrospective design of the study and several known and unknown factors which may have affected the results were not controlled. The objective of this study, however, was to offer a realistic clinical profile of TMD patients with migraine symptoms, rather than to compare specific clinical characteristics while controlling for confounding factors. Still, adjusting for such variables in future studies is necessary to more clearly approach the underlying mechanism between TMD and migraine and establish a causal relationship. Additionally, the diagnosis of migraine was based on patient symptoms and did not involve any imaging or further diagnosis by a neurologist. The study’s reliability could have been enhanced by using metrics such as the MIDAS (Migraine Disability Assessment) for diagnosing migraine. Also, future studies should consider evaluating headache symptoms in a longitudinal manner along with the change in TMD symptoms to investigate long-term effects of treatment and establish the bidirectional relationship between migraine and TMD more clearly. Lastly, some patients among those differentiated as non-migraine also reported headache symptoms. Patients differentiated into the non-migraine group may have had concomitant conventional migraine but were in the interictal period causing difficulty in diagnosis of migraine. This type of bias in diagnosing migraine has continuously been and aspect of debate. As a clinical study aiming to provide data related to TMD patients exhibiting migraine symptoms the results are valid due to the standardized diagnostic protocol however, the presence of headache which is not migraine in certain patients should be considered in interpreting the results and future prospective studies should exclude such patients for more accurate comparison between those with and without migraine.

## 5. Conclusions

This study based on a well-defined TMD patient group found that those with migraine symptoms had higher levels of disability and psychological problems, leading to an overall worse response to conventional TMD treatment short-term compared to TMD patients free of migraine. However, long-term benefits from treatment eventually appeared in both groups as improvement of TMD symptoms. Migraine can complicate the management of TMD to some extent, necessitating that clinicians pay special attention to this specific patient group during the diagnostic process and when addressing psychological conditions.
